# Effect of Topiramate on Morphine-induced Conditioned Place Preference (CPP) in Rats: Role of ERK and CREB Proteins in Hippocampus and Cerebral Cortex

**DOI:** 10.22037/ijpr.2019.1100873

**Published:** 2019

**Authors:** Nima Bagherpasand, Soghra Mehri, Mahdieh Jafari Shahroudi, Seyed Meghdad Tabatabai, Ali Khezri, Mohammad Fathi, Khalil Abnous, Mohsen Imenshahidi, Hossein Hosseinzadeh

**Affiliations:** a *Department of Pharmacodynamics and Toxicology, School of Pharmacy, Mashhad University of Medical Sciences, Mashhad, Iran. *; b *Pharmaceutical Research Center, Pharmaceutical Technology Institute, Mashhad University of Medical Sciences, Mashhad, Iran. *; c *Division of Neurocognitive Sciences, Psychiatry and Behavioral Sciences Research Center, Mashhad University of Medical Sciences, Mashhad, Iran.*

**Keywords:** Topiramate, Conditioned place preference, Morphine, CREB, ERK

## Abstract

In this study, the effect of topiramate, as an antiepileptic drug, was evaluated on morphine craving in rats. The conditioned place preference (CPP) test was used for this purpose. Repeated administration of morphine (10 mg/kg, i.p. for 4 days) induced significant CPP. Administration of topiramate (50 and 100 mg/kg, i.p. for 4 days) with each morphine administration decreased the acquisition of morphine-induced CPP. At the next step, the levels of extracellular signal-regulated kinase (ERK), p-ERK, cAMP responsive element binding (CREB), and p-CREB proteins were evaluated in hippocampus and cerebral cortex using western blot analysis. Following the repeated administration of morphine, the level of p-ERK protein markedly enhanced in both tissues, while topiramate could significantly reduce the phosphorylation of ERK in these brain regions. Additionally, the level of CREB and p-CREB proteins did not change in different groups. Memantine as a positive control reduced the acquisition of morphine-induced CPP. Also, memantine significantly decreased the level of p-ERK protein in hippocampus and cerebral cortex. These results demonstrated that topiramate can attenuate the acquisition of morphine-induced CPP in rats. This effect in part can be mediated through down regulation of p-ERK protein in hippocampus and cerebral cortex.

## Introduction

Opioids are potent analgesic agents with efficacious central and peripheral antinociceptive activities. The development of tolerance to opioids and dependence on them are important problems associated with this class of analgesics (1, 2). The repeated administration of opioids induces physiological and psychological dependence. Physiological dependence is mainly due to desensitization and/or down-regulation of opioid receptors. Opioid deprivation leads to a series of somatic complications described collectively as the opioid withdrawal syndrome. These complications include yawning, sweating, rhinorrhea, anxiety, restlessness, insomnia, dilated pupils, chills, tachycardia, hypertension, nausea, vomiting, abdominal cramps, diarrhea, and muscle pain (3). Psychological dependence on opioids, on the other hand, is associated with the central reward system, which is mainly mediated by the mesolimbic dopamine (DA) system (2). The mesocorticolimbic DA system is involved in reinforcing effects of opioids (4, 5). Opiates activate DA neurons in the ventral tegmental area (VTA) via the inhibition of the local GABAergic inhibitory interneurons, which subsequently elevate the DA transmission to the nucleus accumbens (NAc) (6). 

The VTA and NAc receive glutamatergic projections from the prefrontal cortex (PFC) and limbic areas. The release of DA is also regulated by glutamate. It has been exhibited that memantine, a N-methyl-D-aspartate (NMDA) receptor antagonist, inhibits the acquisition of morphine-induced place preference (7). α-Amino-3-hydroxy-5-methyl-4-isoxazolepropionic acid (AMPA) glutamate receptors also have a key role in long-term changes associated with drug addiction (8).

The hippocampus sends projections to many limbic-related regions, especially the NAc. The hippocampus is involved in several context-dependent processes, including fear conditioning, extinction, drug sensitization, and stress and may play a significant role in reinstatement and relapse in drug-abstinent subjects (9). The repeated administration of cocaine induced long-term potentiation (LTP) in hippocampus and suggested that synaptic plasticity mechanisms may be changed in the manner which led to the development of drug addiction (10). cAMP responsive element binding (CREB) protein level in hippocampus significantly elevated following morphine-induced conditioned place preference (CPP). It was suggested that this region of brain may modulate opioid dependence through CREB target genes regulation (11). 

Also, the role of cortex in modulation of morphine-dependent CPP has been considered (12). In cocaine-induced CPP dopaminergic (DAergic) transmission enhanced from the VTA to the medial prefrontal cortex (mPFC) and D1 receptor was stimulated. Also, the activation of mPFC neurons led to the expression of cocaine CPP (13). 

Finally, it has been shown that following destruction of mPFC, tendency to morphine markedly decreased, which confirmed the important role of this region in morphine addiction (14). 

The signaling pathways involved in addiction have been extensively investigated. Extracellular signal-regulated kinase (ERK) is a member of the MAPK family, which is upregulated in different neuronal populations by chronic morphine administration and produce long-lasting changes (15-17). cAMP responsive element binding (CREB) protein is a gene transcription factor involved in various brain neurocircuitry, including the amygdala, NAc, and hippocampus (18). Additionally, an important role of CREB protein in morphine-induced CPP has been considered (19). It has been reported that p-ERK/ERK ratio and p-CREB/CREB ratio were significantly elevated in different brain areas including NAc, amygdala, striatum, and PFC under morphine-induced CPP (20).

Topitamate, an antiepileptic drug, is used for the treatment of seizures, migraine, obesity, and a variety of other psychiatric disorders (21). The effects of topiramate are mediated through several mechanisms. Topiramate acts as a GABA_A_ (γ-aminobutyric acid) receptor agonist through effect on non-benzodiazepine sites of this receptor (22). Topiramate also blocks calcium channels and decreases the activity of voltage-gated sodium channels (23, 24). Blockade of AMPA and kinate glutamate receptors is another suggested mechanism for topiramate (25, 26).

Although topiramate has been trialed for a number of substance-related disorders including alcohol and cocaine dependence, there is sparse data on its effectiveness in the treatment of opioid dependence (27-29). It has been shown that topiramate can alleviate naloxone-precipitated withdrawal jumping in morphine-dependent mice (30).

Therefore, this study was designed to investigate the effects of topiramate on acquisition of morphine preference as measured by the CPP in rats. Signaling pathways through which topiramate exerts its effects are also assessed using evaluation the level of CREB, p-CREB, ERK and p-ERK proteins in hippocampus and cerebral cortex because these regions of brain have important roles in morphine–induced CPP. 

## Experimental

*Animals*

Male Wistar rats weighing 250-300 g were housed in plastic cages maintained at 21° ± 2° C on a 12-hour circadian cycle. The animals had water and food access *ad libitum* before and during the behavioral tests. All experimental procedures were done in accordance with institutional guidelines for laboratory animal care and use, as directed by the Mashhad University of Medical Sciences ″Animal Studies Ethics Committee″ (No. 910593). Each rat was used only once. A total of 42 rats were randomly divided in 7 groups (n = 6 in each group).

*Drugs*

Morphine sulfate was obtained from Daru Pakhsh, Iran and dissolved in normal saline. Topiramate was provided from Soha, Iran and was dissolved in normal saline with minimal amount of DMSO as co-solvent. The ratio of DMSO to saline was 1 to 5. Memantine was obtained from Osveh, Iran and was dissolved in normal saline. All drugs were injected intraperitoneally (i.p.). 

*CPP test*

The CPP test is a well-recognized and most popular method which is used for evaluation the rewarding effects of drugs (31). In place-conditioning paradigm, animals prefer an environment, which associated with drug administration such as opioids. A typical CPP experiment includes two different sets of environmental signs pairing with the stimulus of interest (32). This test consists of three phases including pre conditioning phase, conditioning phase and post-conditioning phase which were explained completely below. Additionally, the apparatus which is used for our research was described as follows. 

*Apparatus*

Identical plexiglass boxes consisting two equal size compartments (30 cm length × 30 cm width × 35 cm height) and a grey central area (15 cm length × 30 cm width × 35 cm height) were used. The compartments were separated by guillotine doors. One compartment had black walls and smooth floor and the other was white with a harsh texture (33).

*Pre conditioning phase*

During the preconditioning phase, the rats had free access to both compartments of the apparatus for 15 min each day for 2 days. On day 3, the time spent by the animals in each compartment was recorded for 15 min. The animals with strong unconditioned aversion or preference (less than 33% or more than 66% of the session time) for any of the compartments were excluded from the study.

*Conditioning phase*

The conditioning phase was carried out on days 4 to 7. On each day, all groups were received normal saline immediately before confinement to the vehicle-paired compartment for 1 h. After a 4-h interval, the animals received drug injections and were confined in the white section, which was drug-paired compartment for 1 h. 

The confinement was done by closing the guillotine door that separated the two compartments (33) . According to the treatment during this phase, the animals were divided into 7 groups (n = 6): 1) Saline + saline (SAL). 2) Saline + 10 mg/kg of morphine (MOR) (34). 3-5) 10 mg/kg of morphine + 10, 50 or 100 mg/kg of topiramate (MOR+TOP10, MOR+TOP50, and MOR+TOP100, respectively) (30). 6) Saline + 100 mg/kg topiramate (TOP100). 7) 10 mg/kg of morphine + 7.5 mg/kg of memantine as positive control (35). All injections were done via the intraperitoneal (i.p.) route.

*Post-conditioning phase*

**Figure1:**
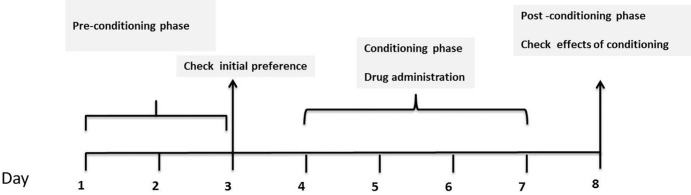
General design of CPP experiment

After conditioning phase, on day 8, each animal was placed in the apparatus, the guillotine door which separated two compartments was removed and the time spent by the animals in each compartment was recorded for 15 min. The time spent in the central area was equally divided between both conditioning compartments (33, 36). Three phases of CPP test have been shown in Figure 1.

*Open field test (OFT)*

At the next step for evaluation locomotor activity, open field test was performed. The apparatus for this test, made of white wood, had a floor of 100 × 100 cm and 30 cm high which divided by lines into 25 squares of 20 × 20 cm. Each rat was placed in the middle of the apparatus, and its behavior was observed for 15 min. The parameters evaluated were the total number of squares crossed (37).

*Tissue sampling*

After behavioral tests, the rats were sacrificed; the brain tissues (cerebral cortex and hippocampus) were dissected and snap-frozen in liquid nitrogen. The samples were stored at −80 °C until use. 

*Western blot analysis*

The samples were homogenized in the buffer containing 50 mM Tris-HCl (pH 7.4), 2 mM EDTA, 2 mM EGTA, 10 mM NaF, 1 mM sodium orthovanadate (Na3VO4), 10 mM β glycerophosphate, 0.2% W/V sodium deoxycholate, 1 mM phenylmethylsulfonyl fluoride (PMSF), and complete protease inhibitor cocktail (Sigma P8340). The homogenized tissue suspension was centrifuged (4 °C, 10 min, 10000 rpm) and the supernatant was collected. The total proteins were separated in 12% SDS-PAGE gels and transferred to polyvinylidene fluoride (PVDF) membranes. 

The blots were blocked with skim milk (5% in TBST) for CREB and ERK proteins. For p-CREB and p-ERK proteins, the blots were blocked with Bovine Serum Albumin (BSA 5% in TBST). Blocking for each protein was done at room temperature for 1.5 h except ERK which was incubated in 4 °C overnight. Rabbit monoclonal anti-serum against CREB (Cell Signaling, USA: #9197), mouse monoclonal anti-serum against p-CREB (Cell Signaling, USA: #9196), rabbit polyclonal anti-serum against P44/42 MAPK (ERK1/2) (Cell Signaling, USA: #9102), mouse monoclonal anti-serum against Phospho-p44/42 MAPK (ERK1/2) (Cell Signaling, USA: #9106), rabbit polyclonal anti-serum against β-actin (Cell Signaling, USA: #4967) or mouse monoclonal anti-serum against β-actin (Cell Signaling, USA: #3700) were used as primary antibodies (diluted 1:1000) and added to PVDF membranes. For CREB, p-CREB and ERK, PVDF membranes were incubated with primary antibodies for 2 h. For p-ERK, membranes were exposed to primary antibody solution for 16 h at 4 °C. Anti-mouse IgG labeled with horseradish peroxidase (Cell Signaling, USA: #7076) and anti-rabbit IgG labeled with horseradish peroxidase (Cell Signaling, USA: #7074) were used as secondary antibodies (1:3000). The membranes were further incubated with secondary antibodies for 2 h at room temperature. Immunoreactive proteins were visualized by a chemiluminescence reaction (Pierce ECL western blotting substrate) and Alliance Gel-doc (Alliance 4.7 Gel doc, UVtec UK). All bands were normalized against β-actin. Quantification of bands density was done by UV Tec software (UK).

*Statistical analysis*

Results are expressed as mean ± SEM for CPP test. Statistical analysis was performed with Two-way analysis of variance (ANOVA), followed by Bonferroni test. For locomotor activity test and western blot analysis One-way ANOVA followed by Tukey-Kramer test was used. Statistical significance was defined as *p* < 0.05.

## Results

*Effects of topiramate on morphine-induced CPP*

**Figure 2:**
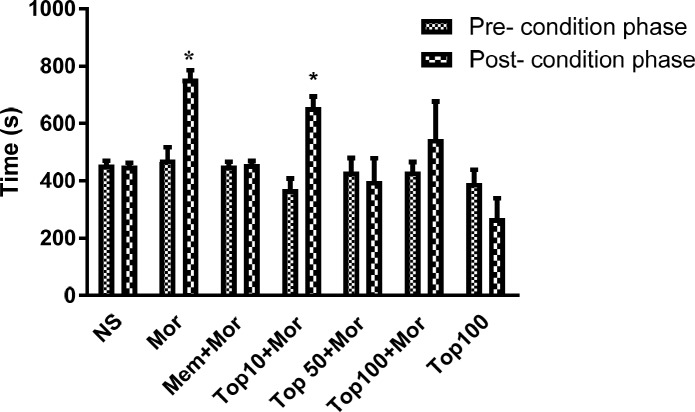
Effects of topiramate (10, 50 and 100 mg/kg) on the acquisition of morphine-induced CPP in rat. During the conditioning phase animals received the different treatments in the drug-paired compartment. Data are expressed as mean ± SEM of 6 animals per group. The bars represent the time spent in the drug-paired compartment before conditioning sessions in pre-conditioning test and after conditioning sessions in post-conditioning test. *p < 0.05 significant differences in the time spent in the drug-paired compartment in pre-conditioning *vs. *post-conditioning sessions tests. NS: Normal saline; Mor: Morphine; Top: Topiramate; Mem: Memantine

As illustrated in Figure 2, repeated administration of morphine (10 mg/kg) for 4 consecutive days induced preference to the morphine-coupled compartment (*p* < 0.05). Saline or topiramate did not exhibit any significant intrinsic rewarding or aversive effects*. *Administration of memantine (7.5 mg/kg) significantly inhibited morphine-induced CPP. Concomitant administration of topiramate (10 mg/kg) with morphine during the conditioning phase did not reverse the preference induced by morphine. However, rats treated with topiramate (50 and 100 mg/kg) did not show any significant preference to the morphine-paired compartment on the post-conditioning phase. There was no significant difference between dose 50 and 100 mg/kg of topiramate on day 8.

*Effect of morphine and topiramate on locomotor activity*

**Figure 3:**
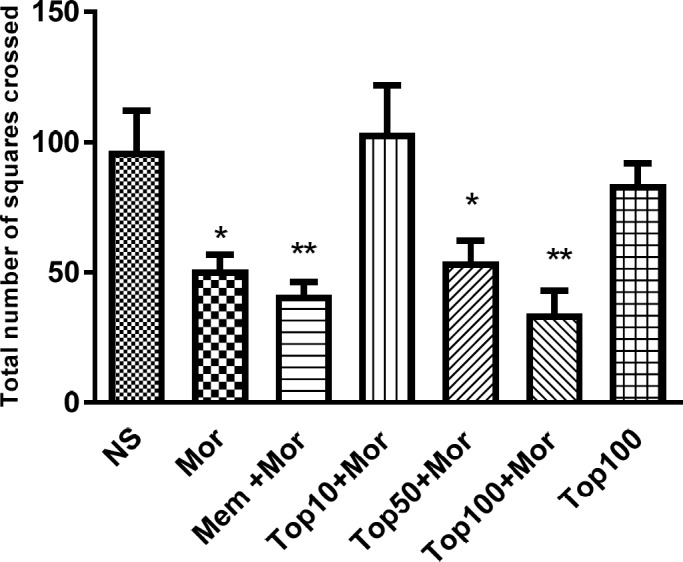
Effect of morphine and topiramate on locomotor activity during open field test in morphine-induced CPP in rat. Bars represent mean ± SEM (n = 6).^ *^p < 0.05 and ^**^p < 0.01 *vs. *NS. The statistical analysis was performed by One-way ANOVA followed by Tukey test. NS: Normal saline; Mor: Morphine; Top: Topiramate; Mem: Memantine

Administration of morphine significantly reduced the number of lines crossed during the open field test as compared to normal saline group (*p* < 0.05). As shown in Figure 3, concomitant administration of topiramate (50 and 100 mg/kg) with morphine markedly decreased locomotor activity in comparison to normal saline group (*p* < 0.05 and *p *< 0.01 respectively). Additionally, treatment of animals with topiramate 100 mg/kg alone did not change locomotor activity when compared to the animals receiving saline. 

*Effect of morphine and topiramate on the level of ERK, p-ERK, CREB and p-CREB proteins in hippocampus and cerebral cortex*

In order to determine the involvement of key signaling molecules in the neurochemical adaptations (associated with the expression of morphine-induced CPP), ERK, p-ERK, CREB and p-CREB proteins were evaluated in two brain regions including hippocampus and cerebral cortex. 

**Figure 4.**
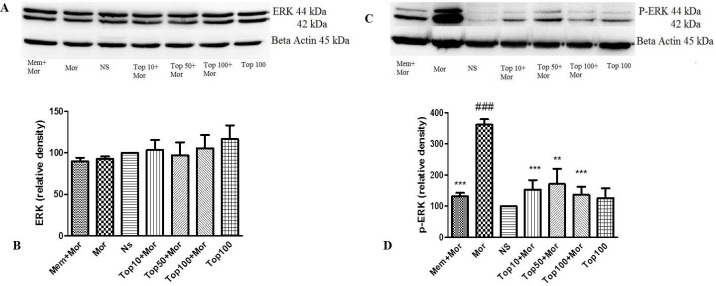
Effect of morphine and topiramate on the protein expression of ERK and p-ERK in hippocampus following morphine-induced CPP in rat. (A and C) Specific bonds of ERK and p-ERK proteins according to western blot analysis. (B and D) Densitometric data of protein analysis. Data are expressed as the mean ± SEM of 4 separate experiments. ANOVA and Tukey-Kramer post-test were used for statistical analysis. ^###^*p *< 0.001 *vs. *NS, ^**^p < 0.01 and ^***^p < 0.001 *vs. *Mor. NS: Normal saline; Mor: Morphine; Top: Topiramate; Mem: Memantine

**Figure 5:**
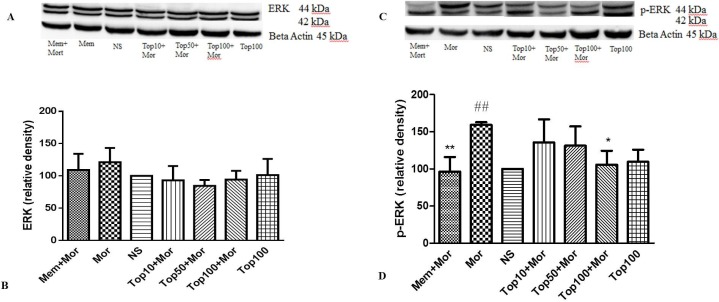
Effect of morphine and topiramate on the protein expression of ERK and p-ERK in cerebral cortex following morphine-induced CPP in rat. (A and C) Specific bonds of ERK and p-ERK proteins according to western blot analysis. (B and D) Densitometric data of protein analysis. Data are expressed as the mean ± SEM of 4 separate experiments. ANOVA and Tukey-Kramer post-test were used for statistical analysis. ^##^*p *< 0.01 *vs. *NS, ^*^p < 0.05 and ^**^p < 0.01 and Mor. NS: Normal saline; Mor: Morphine; Top: Topiramate; Mem: Memantine

**Figure 6:**
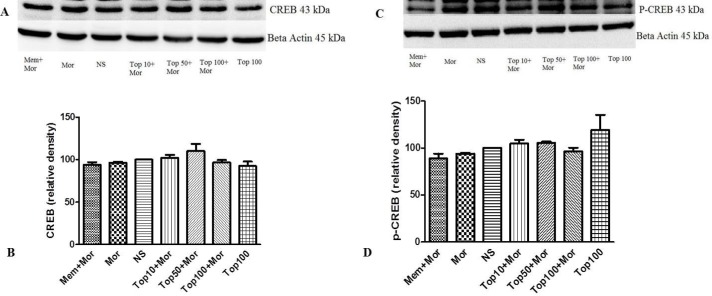
Effect of morphine and topiramate on the protein expression of CREB and p-CREB in hippocampus following morphine-induced CPP in rat. (A and C) Specific bonds of CREB and p-CREB proteins according to western blot analysis. (B and D) Densitometric data of protein analysis. Data are expressed as the mean ± SEM of 4 separate experiments. ANOVA test was used for statistical analysis. NS: Normal saline; Mor: Morphine; Top: Topiramate; Mem: Memantine

**Figure 7:**
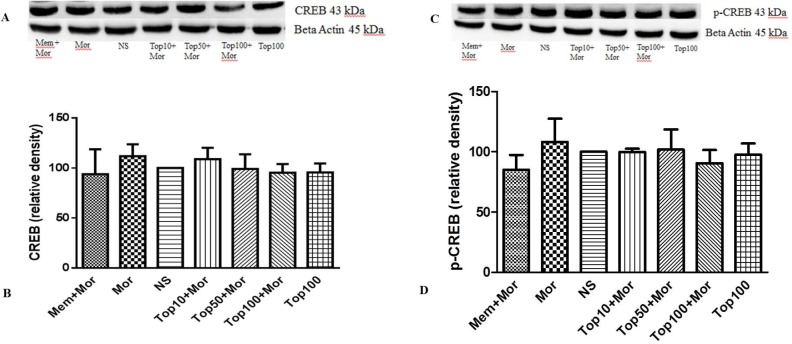
Effect of morphine and topiramate on the protein expression of CREB and p-CREB in cerebral cortex following morphine-induced CPP in rat. (A and C) Specific bonds of CREB and p-CREB proteins according to western blot analysis. (B and D) Densitometric data of protein analysis. Data are expressed as the mean ± SEM of 4 separate experiments. ANOVA test was used for statistical analysis. NS: Normal saline; Mor: Morphine; Top: Topiramate; Mem: Memantine

Our results indicated that treatment of the animals with morphine did not change the levels of ERK (Figures 4A and 4B, Figures 5A and 5B), CREB (Figures 6A and 6B, Figures 7A and 7B) and p-CREB (Figures 6C and 6D, Figures 7C and 7D) in both hippocampus and cerebral cortex tissues when compared to the normal saline group. 

Additionally, administration of different doses of topiramate or memantine (7.5 mg/kg) could not change the level of mentioned proteins. Interestingly, treatment with morphine significantly increased the level of p-ERK protein to 361.9% ± 35.54 and 159.3% ± 3.5 in hippocampus and cerebral cortex, respectively as compared to the normal saline group. Topiramate at different doses (10, 50 and 100 mg/kg) markedly down regulated the level of p-ERK protein in hippocampus, while in cerebral cortex topiramate only at higher dose (100 mg/kg) reversed the level of p-ERK protein (*p* < 0.05 *vs.* morphine). Additionally, co-administration of memantine (7.5 mg/kg) with morphine modulated the level of p-ERK protein in both brain regions (Figures 4C and 4D, Figures 5C and 5D).

## Discussion

Different studies have been performed on the use of topiramate for treatment of substance-related disorders. Nevertheless, the reports about the effect of topiramate on opioid dependence and withdrawal syndrome are very limited (27). In the current study, results demonstrtaed that topiramate is able to inhibit development of morphine-induced CPP in rats. Topiramate did not cause place preference or aversion per se. Additionally administration of morphine elevated the phosphorylation of ERK in cerebral cortex and hippocampus, reversed by topiramate.

It has been shown that topiramate (100 mg/kg) could reduce naloxone-precipitated withdrawal jumping in morphine dependent mice (30).

The roles of intracellular signaling pathways in addiction have been investigated in a large number of studies (16, 38 and 39). The phosphorylation of MAP-kinases and ERK in particular, has been found to be involved in chronic effects of morphine (16) . Wang *et al. *showed that p-ERK content increases in the hippocampus by morphine administration (40). Treatment with ultra-low dose of morphine elevated the level of p–ERK in cortex (41). In our study, the level of ERK protein did not change. However, the phosphorylation of ERK was increased by repeated morphine injection in both hippocampus and cortex tissues. According to the results, administration of topiramate significantly reversed the phosphorylation of ERK in both mentioned brain regions. 

The phosphorylation of ERK results in the activation of a number of signaling molecules including the CREB protein. CREB is a transcription factor expressed in brain cells. The role of CREB in many brain functions has been confirmed. Animal model studies demonstrated that the changes in transcription of CREB mRNA, CREB protein expression and CREB activation may have a key role in psychological disorders such as depression and addiction (39, 42). 

Morphine administration increased CREB activation in locus coeruleus (LC), which, upon chronic administration, undergoes tolerance as a result of inducible CREB expression. Increased CREB phosphorylation has been shown to occur when withdrawal syndrome is precipitated. The blockade of the CREB signaling has been shown to reduce physical signs of morphine withdrawal syndrome. Repeated exposure to opiates or stimulant drugs induces activity of the cAMP–PKA pathway and activate CREB-mediated transcription in the NAc, an important area in development of physical dependence on morphine (18). Increased CREB function in rostral parts of the VTA increases the rewarding effects of cocaine and morphine, whereas similar changes in caudal portions have the opposite effects (43). CREB can increase dynorphin expression which produce aversive or depressive-like effects similar those that often accompany drug withdrawal (44). 

Our results did not show any alterations in the CREB and p-CREB content in the hippocampus and cerebral cortex in morphine-treated groups as compared to the control group. Nevertheless, Zhou *et al.* showed that CREB expression increased in the conditioning phase in the hippocampus of rats (11). In another study CREB phosphorylation markedly elevated in the hippocampus following ohmefentanyl (a mu opioid agonist) induced place preference (45). Also, CREB phosphorylation was shown to increase in morphine-induced place preference in the hippocampus and the cortex (12). 

Possible explanations for different results may be related to this fact that in our study the conditioning phase was short, also the enhancement of p-ERK protein may be was not enough sufficient to elevate the CREB and p-CREB protein levels. It has been reported that blockade of AMPA receptors in the LC suppresses the behavioral signs of morphine withdrawal signs (8). A selective AMPA antagonist also inhibited behavioral morphine sensitization (46). The overexpression of GluR1, (an AMPA subunit) in the VTA of the rats also increased morphine sensitization, supporting a role for AMPA receptors in locomotor and motivational adaptations in morphine dependence (47). The phosphorylation of GluR1 is also increased in morphine conditioned place preference in the hippocampus and cortex associated with learning and memory (12). Topiramate exhibited antagonistic effects on AMPA and kinate glutamate receptors (25, 26). Therefore, it is speculated that another major mechanism of topiramate suppressing place preference is blockade of AMPA receptors, owing especially to the fact that GluR1 phosphorylation (activation) was concomitant with CREB and ERK phosphorylation during the drug sensitization and conditioning in a number of studies (48).

In conclusion, our study exhibited that topiramate is able to reverse the CPP induced by morphine without having any rewarding or aversive effects by itself. Topiramate may exert this effect by down regulation of intracellular signaling pathways as it decreased p-ERK content in the hippocampus and cortex. Additionally, other mechanisms including AMPA glutamate antagonism and GABA_A _modulation may be involved. Regarding to potent effects of topiramate in reduction of morphine-induced CPP, further clinical studies are suggested for evaluation topiramate effect for the treatment of addiction and substance craving.
